# Free thyroxine level is associated with both relapse rate and poor neurofunction in first-attack Neuromyelitis Optica Spectrum Disorder (NMOSD) patients

**DOI:** 10.1186/s12883-019-1560-7

**Published:** 2019-12-18

**Authors:** Qianyi He, Lifeng Li, Yanfei Li, Yanhui Lu, Kaimin Wu, Ruiyi Zhang, Junfang Teng, Jie Zhao, Yanjie Jia

**Affiliations:** 1 Department of Neurology, the First Affiliated Hospital of Zhengzhou University, Zhengzhou, 450052 Henan China; 2 Department of Pharmacy, the First Affiliated Hospital of Zhengzhou University, Zhengzhou, Henan China; 3 National Telemedicine Center, the First Affiliated Hospital of Zhengzhou University, Zhengzhou, Henan China; 4 Internet Medical and System Applications of National Engineering Laboratory, Zhengzhou, Henan China

**Keywords:** Serum free thyroxine, NMOSD, Prognosis, Relapse, EDSS

## Abstract

**Background:**

To investigate whether the serum free thyroxine (FT4) level is a prognostic factor for the first-attack neuromyelitis optica spectrum disorders (NMOSD).

**Methods:**

This retrospective study enrolled 109 patients with first-attack NMOSD. The Expanded Disability Status Scale (EDSS) and the relapse rate were used to evaluate the outcomes. The logistic regression model was used to analyze the independent effects of FT4 on relapse and final EDSS. Kaplan-Meier analysis, scatter plot smoothing method, and two-phase piecewise linear regression model were used to investigate the relationship between the FT4 level and the relapse rate.

**Results:**

Multivariate analysis revealed that serum FT4 level might be a risk factor for both final EDSS (β = 0.17; 95% confidence interval: 0.03–0.32) and the relapse rate (HR = 1.18; 95% confidence interval: 1.05–1.32). Furthermore, 1400 days after the onset, nearly 100% of patients in the high-FT4 group relapsed, while only 40% of the patients in the low-FT4 group relapsed. Finally, we found that the relationship between the FT4 level and the NMOSD relapse rate was nonlinear. The risk of NMOSD relapse increased with the FT4 level up to the inflection point of 12.01 pmol/L (HR = 1.45; 95% confidence interval: 1.06–1.98). When the FT4 level was > 12.01 pmol/L, there was no correlation between the FT4 level and the risk of NMOSD relapse (HR = 1.05; 95% confidence interval: 0.78–1.41).

**Conclusion:**

Serum FT4 level may be a prognostic indicator for the first-attack in patients with NMOSD. High FT4 levels are associated with poor neurofunctions and a high relapse rate in patients with the first-attack in patients with NMOSD.

## Background

Neuromyelitis optica spectrum disorders (NMOSD) represent a group of autoimmune diseases that are characterized by inflammation and demyelination of the optic nerve, spinal cord and central nervous system [1]. Historically, NMOSD was considered to be a subtype of multiple sclerosis. Since the discovery of NMO-IgG, an NMO-specific autoantibody directed against aquaporin-4 (AQP-4), neurologists have identified NMOSD as a distinct neuropathy from multiple sclerosis [2]. In contrast with multiple sclerosis, a greater degree of the disability associated with NMOSD is due to severe optic nerve and spinal cord impairment and this condition responds poorly to immunomodulatory therapies [1, 3]. Moreover, it has been widely accepted that there is variability in the severity of NMOSD across patients [4]. Therefore, the identification of reliable and sensitive biomarkers for predicting the prognosis of NMOSD is important.

Thyroid hormones have a wide and important range of effects within the nervous system beginning from fetal life and continuing throughout the adult life [5, 6]. Thyroid dysfunction is common in autoimmune diseases of the nervous system, especially in neurological demyelinating disorders such as acute transverse myelitis, Guillain-Barré syndrome and multiple sclerosis [7–12]. Patients with acute transverse myelitis have lower levels of thyroid stimulating hormones (TSH) and free triiodothyronine (FT3) and higher levels of free thyroxine (FT4) and FT4/FT3 ratio than in healthy controls [7]. A higher FT4 level and lower TSH level may be associated with the incidence and severity of Guillain-Barré syndrome [8]. In cerebrospinal fluid, the total T4 level (TT4) and the TT4/TT3 ratio in patients with multiple sclerosis are significantly higher than those in normal controls [11].

A previous study showed that a low FT3 level may indicate a poor prognosis of NMOSD [9]. Additionally, compared with multiple sclerosis, in NMOSD there is a higher frequency of abnormal levels of TSH, anti-thyroglobulin antibodies and antithyroid peroxidase antibodies than in other conditions [10]. However, the relationship between serum FT4 and NMOSD is still unknown. The aim of this study was to investigate the correlation between the level of FT4 and the prognosis of NMOSD patients following their first attack.

## Methods

### Participants

**Fig. 1:**
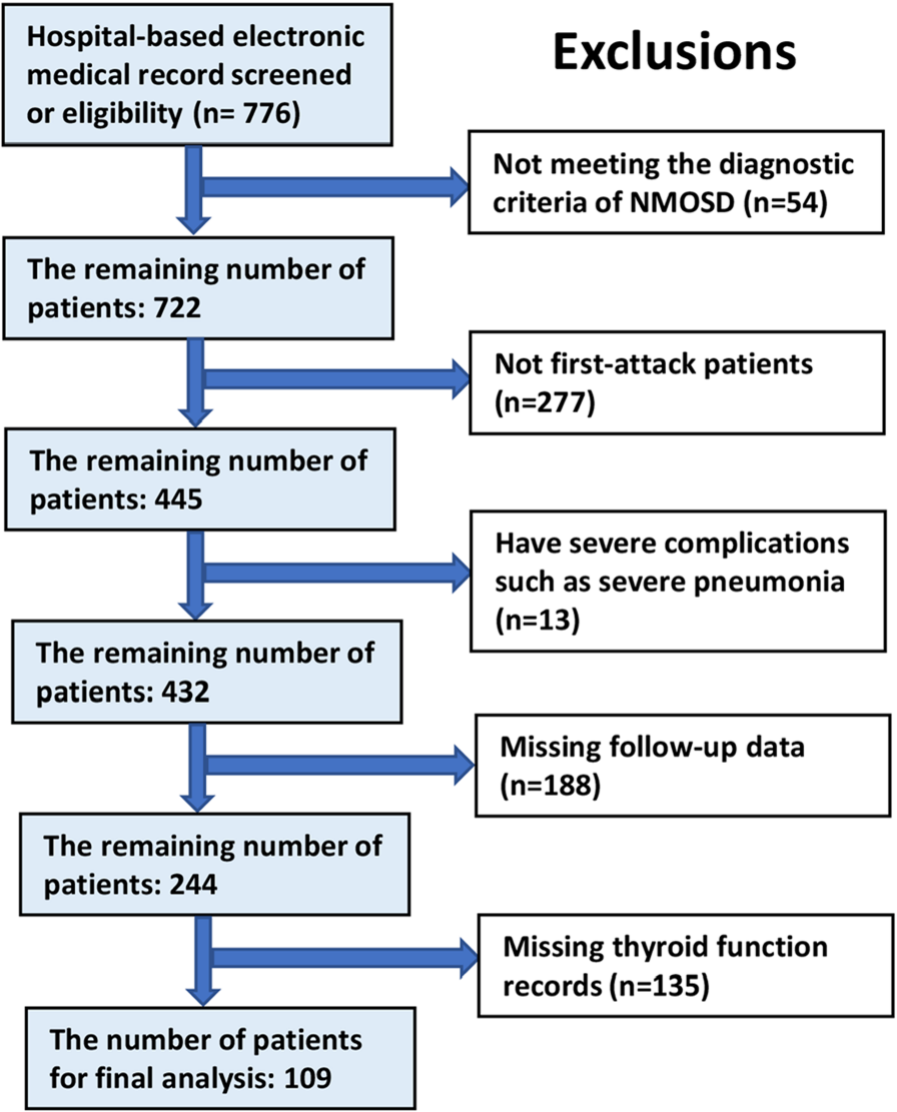
Flowchart of participant selection

In this retrospective study, we collected the clinical data of 244 patients following their first-attack of NMOSD from the First Affiliated Hospital of Zhengzhou University between June 2012 and March 2018. NMOSD was diagnosed according to the Wingerchuk 2006 criteria or the 2015 international consensus diagnostic criteria for NMOSD [13, 14]. All attacks of NMOSD in the identified patients were assessed and only patients that had experienced only one attack of NMOSD were enrolled in this study. The exclusion criteria were as follows: (1) follow-up data or thyroid function results were missing; (2) a history of conditions that affect thyroid function, such as Hashimoto’s thyroiditis, thyroidectomy, hypothalamus disease and pituitary disease; (3) coexistence of other neurologic or ophthalmic diseases that may affect the EDSS; (4) sequelae of ophthalmic or neurologic diseases; (5) coexistence of affective disorders, malignant diseases, substance abuse or confusion. A total of 109 patients were eventually enrolled. A flowchart of participant selection is shown in Fig. 1. The project was approved through the Ethics Committee of Zhengzhou University (Ethics review number: 2019-KY-018).

### Data collection

The individual clinical data were collected, including gender, age at onset, previous medical history, treatment for NMOSD and thyroid function parameters (FT3, FT4, TSH).

Follow-up data were obtained through an annual clinic visit or telephone interview every 3 months. The severity of NMOSD and outcomes were assessed independently by two experienced neurologists, according to Expanded Disability Status Scale (EDSS) at the initial presentation (initial EDSS) and at the final follow-up (final EDSS), respectively. Relapse was defined as new-onset, progressive, or recurrent neurological symptoms caused by a central nervous system demyelinating disease, which lasted for at least 24 h and led to an EDSS increase of at least 0.5 points than the lowest score. The main prognostic parameters in this study were the final EDSS score and the relapse rate of NMOSD.

FT3 (reference range, 3.28–6.47 pmol/L) and FT4 (reference range, 7.9–18.4 pmol/L) levels were measured using a commercial radioimmunoassay (RIA) kit (Roche Diagnostics, Mannheim, Germany). TSH (reference range, 0.34–5.6 μIU/mL) levels were measured using a commercial RIA kit (Immunotech, Marseille, France).

### Statistical analysis

All statistical analyses were performed using R packages (http://www.R-project.org; TeRFoundation) and EmpowerStats software. Continuous and categorical variables are presented as mean ± standard deviation (SD) and percentage, respectively. Continuous variables were compared using *t* tests when the data were normally distributed or Kruskal-Wallis rank sum tests when the data were non-normally distributed. Spearman correlation analysis was performed between the FT4 level and the initial and final EDSS score changes from baseline to the first treatment. Categorical data were compared using the chi-square test. Univariate logistic regression was used to identify the potential risk factors. Multivariate logistic regression model was used to analyze the independent effect of FT4 on the relapses rate and final EDSS. Kaplan-Meier analysis of the accumulation of NMOSD relapse was performed. The correlation between FT4 and NMOSD relapse was analyzed through the scatter plot smoothing method, and the threshold effect of FT4 on relapse was investigated using a two-phase piecewise linear regression model. Probability (*p*) values ≤0.05 were considered significant.

## Results

A total of 109 patients with first-attack NMOSD were enrolled in this study. These patients were divided into a low-FT4 group (with serum FT4 level < the median level; *n* = 54) and a high-FT4 group (serum FT4 level > the median level; *n* = 55). There were significant differences in the levels of hypertension (5.56% vs. 18.18%; *p* = 0.042), diabetes (0.00% vs. 12.73%, *p* = 0.007), anti-AQP-4 antibodies (31.8% vs. 58.18%, *p* = 0.009), and the relapse rate (31.48% vs. 67.27%, *p* < 0.001) between these two groups. The demographic and clinical characteristics are summarized in Table 1.

**Table 1:** Demographics and clinical characteristics of the patients

Characteristics	Low-FT4 group (*n* = 54)	High-FT4 group (*n* = 55)	*P* value
Age -years	41.21 ± 13.78	43.18 ± 17.00	0.508
Male sex- no. (%)	16 (29.63)	11 (20.00)	0.244
Hypertension - no. (%)	3 (5.56)	10 (18.18)	0.042^*^
Diabetes - no. (%)	0 (0.00)	7 (12.73)	0.007^*^
Initial EDSS	5.03 ± 1.73	4.75 ± 1.87	0.415
Vision (EDSS of Vision)	1.85 ± 2.10	1.56 ± 1.86	0.451
Optic neuritis - no. (%)	13 (24.07)	17 (30.91)	0.559
Acute myelitis - no. (%)	38 (70.37)	35 (63.64)	0.587
LESCL - no. (%)	40 (74.07)	42 (76.36)	0.956
Anti-AQP-4 antibody - no. (%)			0.009^*^
Not tested	21 (38.89)	9 (16.36)	
Negative	16 (29.63)	14 (25.45)	
Positive	17 (31.48)	32 (58.18)	
Autoimmune - no. (%)	2 (3.70)	3 (5.45)	0.662
Corticosteroid - no. (%)	48 (88.89)	47 (85.45)	0.592
IVIG - no. (%)	2 (3.70)	3 (5.45)	0.662
Immunosuppressant - no. (%)	16 (29.63)	10 (18.18)	0.161
Rehabilitation - no. (%)	13 (24.07)	10 (18.18)	0.451
FT3 - pmol/L	4.48 ± 0.66	4.61 ± 0.98	0.447
FT4 - pmol/L	9.55 ± 1.45	13.33 ± 1.78	< 0.001^*^
TSH -μIU/mL	4.75 ± 13.16	1.70 ± 1.33	0.090
Interval - days	592.72 ± 423.23	472.78 ± 364.31	0.116
Final EDSS	1.37 ± 1.74	1.76 ± 1.79	0.249
Relapse - no. (%)	17 (31.48)	37 (67.27)	< 0.001^*^

*no.* Number, *EDSS* Expanded Disability Status Scale, *LESCL* Longitudinally extensive spinal cord lesions, *IVIG* Intravenous immunoglobulin, *FT3* Free triiodothyronine, *FT4* Free thyroxine, *TSH* Thyroid stimulating hormone

^*^P < 0.05 indicates a significant difference between the two groups

**Fig. 2:**
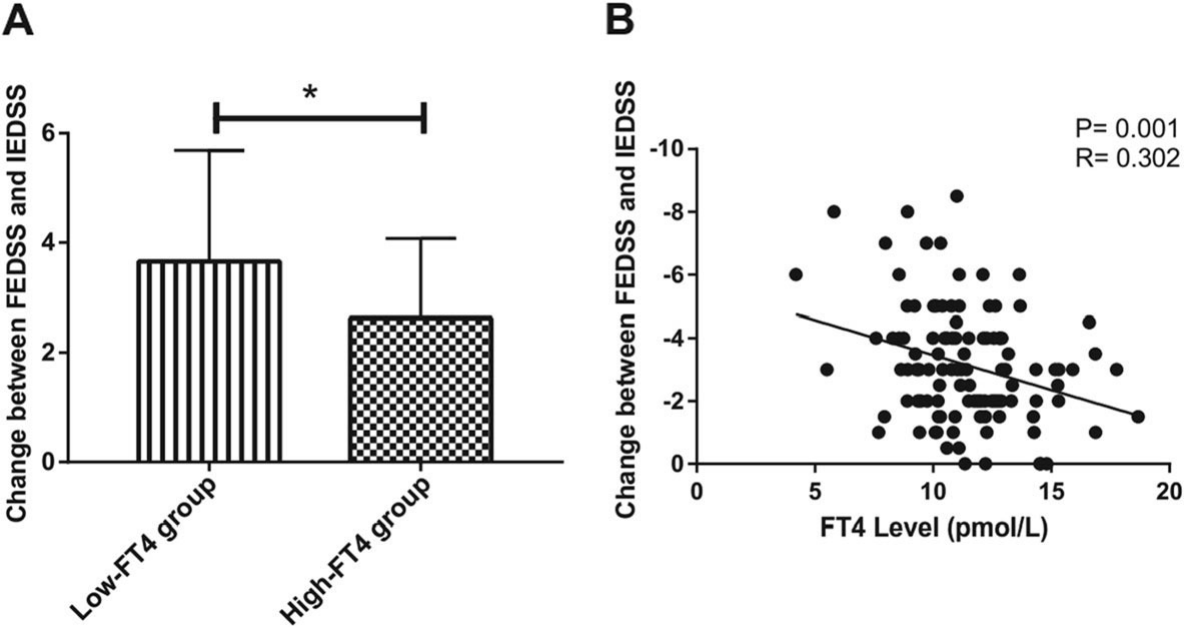
The FT4 level correlates with changes between FEDSS and IEDSS. **a** Changes between FEDSS and IEDSS in the low-FT4 group (n = 54) and the high-FT4 group (*n* = 55). Values are presented as “mean ± standard deviation”. **b** The FT4 level is negatively correlated with changes between FEDSS and IEDSS (*n* = 109)

Univariable analysis showed that hypertension (HR = 2.76; 95% confidence interval: 1.37–5.56) and serum FT4 level (HR = 1.16; 95% confidence interval: 1.04–1.28) were significantly associated with the relapse rate, and that the initial EDSS (β = 0.36; 95% confidence interval: 0.19–0.53) and serum FT4 level (β = 0.19; 95% confidence interval: 0.06–0.32) were positively related to the final EDSS scores. The serum FT4 level was correlated with both the relapse rate and the final EDSS scores. Detailed statistical data are presented in Table 2. The relationship between the FT4 level and the EDSS changes (FEDSS minus IEDSS in the same patient), which represents the improvement of NMOSD symptoms, were then analyzed. We found that the low-FT4 group had larger EDSS changes than the high FT4 group (Fig. 2a). Moreover, EDSS changes were negatively related with the FT4 level (Fig. 2b).

**Table 2:** Univariable analysis of potential the risk factors for predicting the prognosis of NMOSD

Factor	Statistical value	Relapse		Final EDSS	
		HR (95% CI)	*P* value	β (95% CI)	*P* value
Male sex - no. (%)	27 (24.77)	0.71 (0.35, 1.42)	0.331	0.06 (−0.72, 0.83)	0.887
Age (years)	42.21 ± 15.45	1.00 (0.99, 1.02)	0.699	0.00 (− 0.02, 0.03)	0.754
Hypertension - no. (%)	13 (11.93)	2.76 (1.37, 5.56)	0.004^*^	−0.17 (−1.20, 0.87)	0.754
Diabetes - no. (%)	7 (6.42)	1.64 (0.65, 4.14)	0.293	−0.15 (− 1.51, 1.21)	0.830
Initial EDSS	4.89 ± 1.80	0.95 (0.82, 1.11)	0.536	0.36 (0.19, 0.53)	< 0.001^*^
Vision (EDSS of vision)	1.71 ± 1.98	1.05 (0.91, 1.21)	0.491	0.01 (−0.15, 0.18)	0.866
Anti-AQP-4 antibody - no. (%)					
Not tested	30 (27.52)	1.0			
Negative	30 (27.52)	0.72 (0.33, 1.57)	0.413	0.03 (−0.86, 0.92)	0.942
Positive	49 (44.95)	1.20 (0.64, 2.23)	0.569	0.69 (−0.11, 1.49)	0.094
Autoimmune - no. (%)	5 (4.59)	1.98 (0.61, 6.41)	0.256	0.56 (−1.04, 2.15)	0.495
Corticosteroid - no. (%)	95 (87.16)	1.06 (0.51, 2.21)	0.881	0.53 (−0.46, 1.52)	0.299
IVIG - no. (%)	5 (4.59)	0.91 (0.22, 3.73)	0.891	1.29 (−0.29, 2.87)	0.112
Immunosuppressant - no. (%)	26 (23.85)	1.41 (0.76, 2.62)	0.272	−0.42 (−1.20, 0.36)	0.295
Rehabilitation - no. (%)	23 (21.1)	1.24 (0.65, 2.37)	0.510	0.19 (−0.63, 1.01)	0.653
FT3 - pmol/L	4.55 ± 0.83	0.96 (0.69, 1.34)	0.827	0.20 (−0.21, 0.60)	0.343
FT4 - pmol/L	11.46 ± 2.49	1.16 (1.04, 1.28)	0.006^*^	0.19 (0.06, 0.32)	0.006^*^
TSH -μIU/mL	3.21 ± 9.39	0.98 (0.93, 1.03)	0.419	−0.02 (−0.06, 0.01)	0.216

*no.* Number, *EDSS* Expanded Disability Status Scale, *IVIG* Intravenous immunoglobulin, *FT3* Free triiodothyronine; *FT4* Free thyroxine, *TSH* Thyroid stimulating hormone

^*^P < 0.05 indicates a significant difference between the two groups

The multivariate logistic regression model was used to examine the independent effect of FT4 on the relapses rate and final EDSS score. Covariate screening was analyzed using the computer software. For the first model, the screening criteria included risk factors producing > 10% changes in the regression coefficient after introduction into the basic model. For the second model, besides the covariates included in the first model, we also included the factors that have potential interactions with the FT4 level or the outcomes. After the adjustment of the covariates affecting the relationship between FT4 and relapse rate or final EDSS, serum FT4 level were still a risk factor for both NMOSD relapse rate (Table 3, model 1: HR = 1.18; 95% confidence interval: 1.05–1.32; model 2: HR = 1.19; 95% confidence interval: 1.05–1.35) and the final EDSS scores (Table 4, model 1: β = 0.16; 95% confidence interval: 0.03–0.30; model 2: β = 0.17; 95% confidence interval: 0.03–0.32).

**Table 3:** Correlation analysis between FT4 and relapse rate

Exposure	Non-adjusted	*P* value	Adjust I	*P* value	Adjust II	*P* value
FT4	1.17 (1.05, 1.30)	0.006	1.18 (1.05, 1.32)	0.005	1.19 (1.05, 1.35)	0.005

In the adjust I model, the adjusted variables include FT3, hypertension, diabetes, and immunosuppressant;

In the adjust II model, the adjusted variables include FT3, anti-AQP-4 antibody, TSH, gender, age, initial EDSS, hypertension, diabetes, autoimmune, corticosteroid, IVIG, and immunosuppressant

**Table 4:** Correlation analysis between FT4 and final EDSS

Exposure	Non-adjusted	*P* value	Adjust I	*P* value	Adjust II	*P* value
FT4	0.19 (0.06, 0.32)	0.006	0.16 (0.03, 0.30)	0.015	0.17 (0.03, 0.32)	0.013

In the adjust I model, the adjusted variable was anti-AQP-4 antibody;

In the adjust II model, the adjusted variables include FT3, anti-AQP-4 antibody, TSH, gender, age, initial EDSS, hypertension, diabetes, autoimmune, corticosteroid, IVIG, and immunosuppressant

**Fig. 3:**
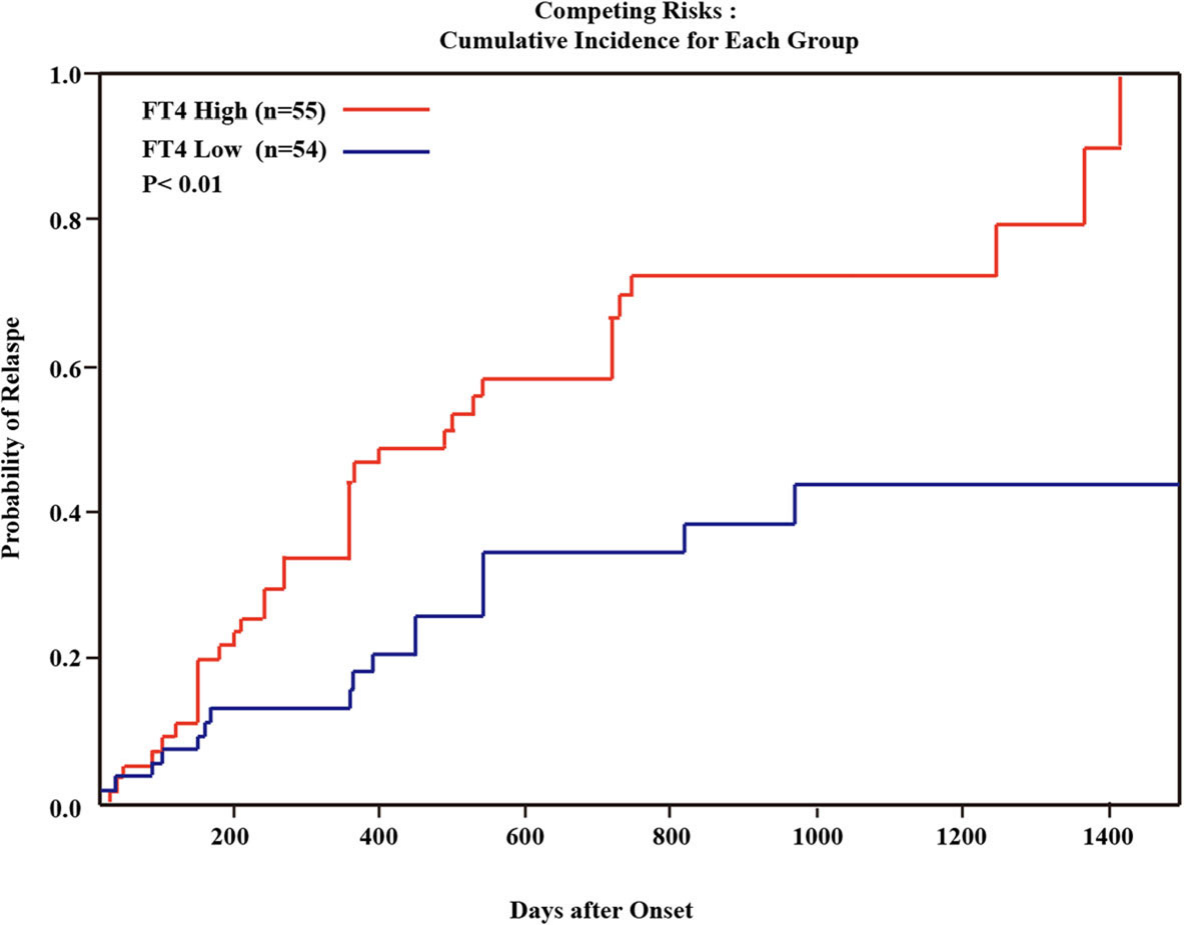
Kaplan-Meier analysis showing the cumulative NMOSD relapse rate in the low-FT4 group and the high-FT4 group

The correlation between the FT4 level and the cumulative NMOSD relapse rate was then evaluated using Kaplan-Meier analysis. The median interval to relapse was 481 days in the high-FT4 group and > 1400 days in the low-FT4 group, while nearly 100% FT4-high group patients relapsed 1400 days after attack (Fig. 3).

**Fig. 4:**
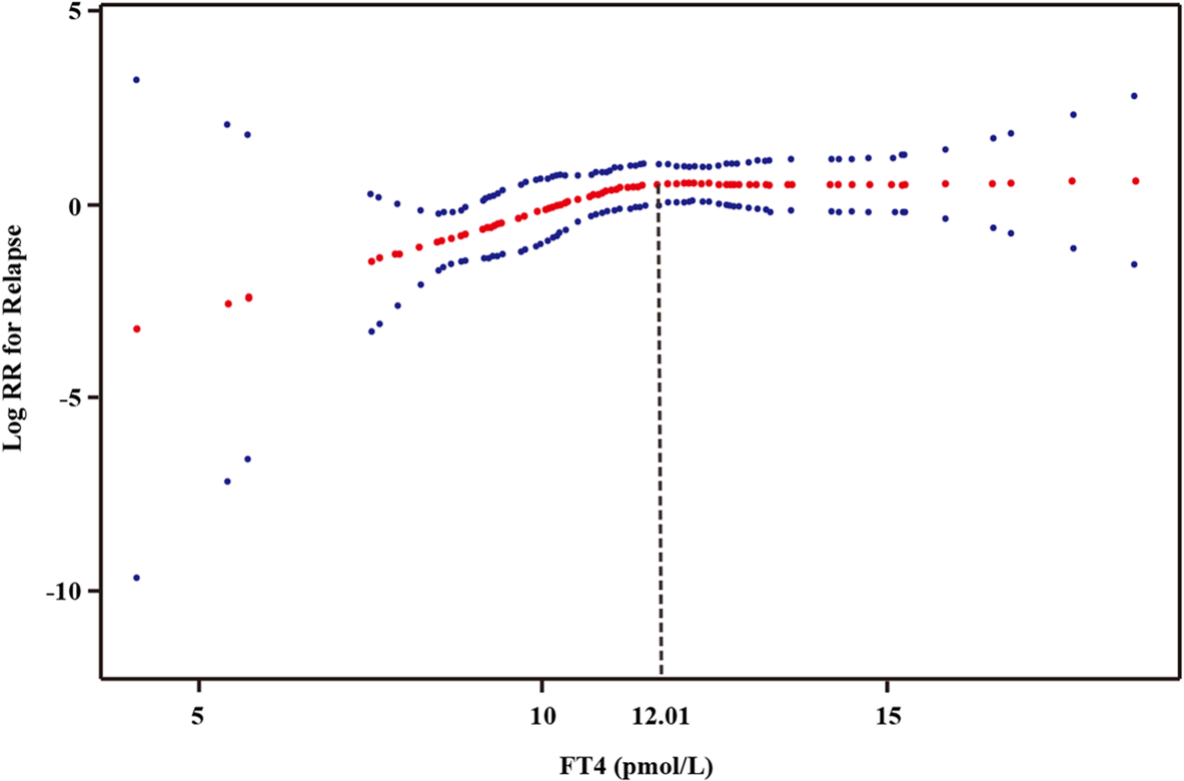
Smoothed scatter plot showing the correlation between the serum FT4 level and the risk of NMOSD relapse. The inflection point of FT4 level was 12.01 pmol/L

After adjustment of the possible factors related to NMOSD relapse and serum FT4 level, including serum FT3 level, serum TSH level, age, gender, anti-AQP-4 antibody, initial EDSS scores, corticosteroid administration, intravenous immunoglobulin administration, and immunosuppressant administration, a nonlinear relationship was observed between the serum FT4 level and the risk of NMOSD relapse (Fig. 4). There was a correlation between the risk of NMOSD relapse and the FT4 level up to the inflection point of 12.01 pmol/L (HR = 1.45; 95% confidence interval: 1.06–1.98). When the FT4 level was > 12.01 pmol/L, there was no correlation between the FT4 level and the risk of NMOSD relapse (HR = 1.05; 95% confidence interval: 0.78–1.41).

## Discussion

To the best of our knowledge, this is the largest single-center study to investigate the relationship between the serum FT4 level and the prognosis of first-attack NMOSD patients. We found that a high FT4 level may be a risk factor for both relapse and poor EDSS performance in patients with NMOSD. Furthermore, we found that the relationship between FT4 level and NMOSD relapse rate was nonlinear. The infection point of FT4 level was 12.01 pmol/L.

In the literature, there are some multi-center studies on the prognosis of NMOSD [15–18]. There may be some advantages to these multi-center studies, such as the fact that the sample sizes are relatively large and the findings can be more generally applicable. However, the multi-center design requires more strict quality control and it may have potential measurement bias. In our study, the EDSS scores were assessed independently by two experienced neurologists and conflicts were solved thorough discussion. Unlike previous single-center studies on the prognosis of NMOSD, [19–29] we focused on the first-attack of NMOSD, which enabled us to rule out the effects of previous treatment (such as glucocorticoids, rituximab and immunosuppressants). The mean follow-up period in this study was 17.7 months. According to a previous report, [4] the median time to the first relapse in patients with NMOSD is 14 months; thus, our follow-up time was sufficient to draw relevant conclusions.

The pathogenetic mechanism of thyroid dysfunction in NMOSD remains unclear. We speculate there may be two main causes: 1) thyroid hormones enhance the immune response, which causes damage of the NMOSD. Since the discovery of the anti-AQP-4 antibody, NMOSD has been considered to be driven by humoral immunity mechanisms. Previous studies have indicated that L-thyroxine enhances the phenotype percentage and function of human peripheral blood dendritic cells, which are the most effective antigen-presenting cells and are key regulators of immune response [30]. Furthermore, B cells can also be regulated by thyroid hormones, as thyroid hormones positively regulate primary B cell lymphopoiesis [31]. In-vivo treatment of thyroid hormone-deficient mice with T4 increases both the percentage and total number of circulating pro-B cells, suggesting that thyroid hormones can regulate the proliferative potential of developing B cell precursors [32]. 2) When the serum FT4 level exceeds the inflection point (12.01 pmol/L), more thyroid hormones can penetrate the blood brain barrier and play a neuroprotective role. Thyroid hormone receptors are distributed widely in the nervous system and are important for the regulation of growth and myelination [33, 34]. It has been proven that thyroid hormones can alleviate multiple sclerosis by promoting remyelination, which may be attributed to the regulatory role of FT4 in the gene transcription of oligodendrocyte precursor cells [35]. In the cerebrospinal fluid, the reverse triiodothyronine (rT3) levels or TT4/rT3 ratio are positively associated with the level of nerve growth factor [11]. However, further research is still required to draw a definitive conclusion.

There are some limitations to this study. First, the retrospective design has inherent defects. Second, previous treatment and concomitant disorders could not be thoroughly assessed. This might have a potential influence on the prognosis. Additionally, the serum levels of thyroid hormones (FT4, FT3 and TSH) were only available for a fraction of our patients (109/244), which may yield a selection bias. Finally, the levels of anti-AQP-4 antibodies were not examined in all of the included patients (Our hospital was unable to test for anti-AQP-4 antibodies before June 2013).

## Conclusion

Serum FT4 level may be a prognostic indicator for first-attack NMOSD. A high FT4 level is associated with a poor neurofunction and a high NMOSD relapse rate. The study is preliminary and more data are needed to make conclusions.

## Data Availability

All data are available without restriction from corresponding author on reasonable request.

## Abbreviations

AQP-4: Aquaporin-4
EDSS: Expanded Disability Status Scale
FT3: Free triiodothyronine
FT4: Free thyroxine
HR: Hazard ratio
IVIG: Intravenous immunoglobulin
NMO: Neuromyelitis optica
NMOSD: Neuromyelitis optica spectrum disorders
RIA: Radioimmunoassay
rT3: reverse triiodothyronine
SD: Standard deviation
TSH: Thyroid stimulating hormones
TT3: Total triiodothyronine
TT4: Total thyroxine
